# (Ferrocenylmeth­yl)dimethyl­ammonium bromide

**DOI:** 10.1107/S1600536810018453

**Published:** 2010-05-22

**Authors:** Bo Wang

**Affiliations:** a Ordered Matter Science Research Center, Southeast University, Nanjing 210096, People’s Republic of China

## Abstract

The title compound, [Fe(C_5_H_5_)(C_8_H_13_N)]Br, is isotypic with the analogous chloride compound. The Fe—C bond lengths are in the range 2.020 (6)–2.048 (7) Å. In the crystal, the cations and bromide anions are connected by N^+^—H⋯Br^−^ hydrogen bonds.

## Related literature

For the isotypic chloride compound, see: Winter & Wolmershauser (1998 ▶). For other structures containing the (*N*-ferrocenylmeth­yl)dimethyl­ammonium cation, see: Gibbons & Trotter (1971 ▶); Guo (2006 ▶); Guo, Yang & Zhang (2006 ▶); Guo, Zhou *et al.* (2006 ▶).
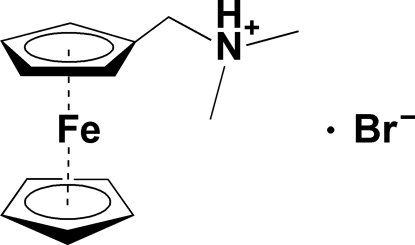


## Experimental

### 

#### Crystal data

[Fe(C_5_H_5_)(C_8_H_13_N)]Br
*M* *_r_* = 324.04
Orthorhombic, 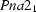

*a* = 21.393 (4) Å
*b* = 5.9296 (12) Å
*c* = 10.798 (2) Å
*V* = 1369.7 (5) Å^3^
*Z* = 4
Mo *K*α radiation
μ = 3.99 mm^−1^
*T* = 298 K
0.20 × 0.20 × 0.20 mm

#### Data collection

Rigaku SCXmini diffractometer
Absorption correction: multi-scan (*CrystalClear*; Rigaku, 2005 ▶) *T*_min_ = 0.450, *T*_max_ = 0.468
13217 measured reflections
3117 independent reflections
2638 reflections with *I* > 2σ(*I*)
*R*_int_ = 0.056

#### Refinement

*R*[*F* ^2^ > 2σ(*F* ^2^)] = 0.038
*wR*(*F* ^2^) = 0.078
*S* = 1.08
3117 reflections
147 parameters
1 restraint
H-atom parameters constrained
Δρ_max_ = 0.28 e Å^−3^
Δρ_min_ = −0.51 e Å^−3^
Absolute structure: Flack (1983 ▶), 1474 Friedel pairs
Flack parameter: 0.011 (12)

### 

Data collection: *CrystalClear* (Rigaku, 2005 ▶); cell refinement: *CrystalClear*; data reduction: *CrystalClear*; program(s) used to solve structure: *SHELXS97* (Sheldrick, 2008 ▶); program(s) used to refine structure: *SHELXL97* (Sheldrick, 2008 ▶); molecular graphics: *SHELXTL* (Sheldrick, 2008 ▶); software used to prepare material for publication: *PRPKAPPA* (Ferguson, 1999 ▶).

## Supplementary Material

Crystal structure: contains datablocks I, global. DOI: 10.1107/S1600536810018453/bi2381sup1.cif

Structure factors: contains datablocks I. DOI: 10.1107/S1600536810018453/bi2381Isup2.hkl

Additional supplementary materials:  crystallographic information; 3D view; checkCIF report

## Figures and Tables

**Table 1:** Hydrogen-bond geometry (Å, °)

*D*—H⋯*A*	*D*—H	H⋯*A*	*D*⋯*A*	*D*—H⋯*A*
N1—H1*A*⋯Br1	0.91	2.28	3.172 (3)	165

**Fig. 1.**
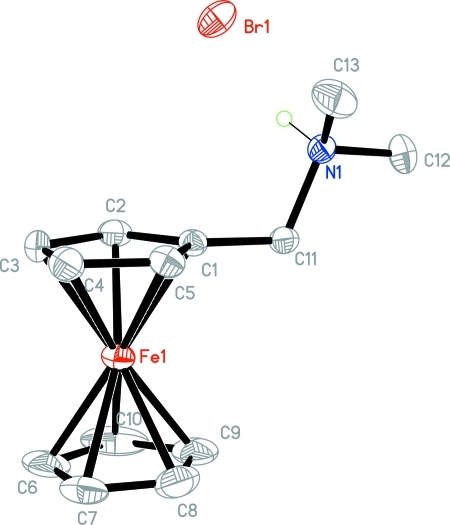
Molecular structure of the title compound, with displacement ellipsoids drawn at the 30% probability level. H atoms on C have been omitted for clarity.

**Fig. 2.**
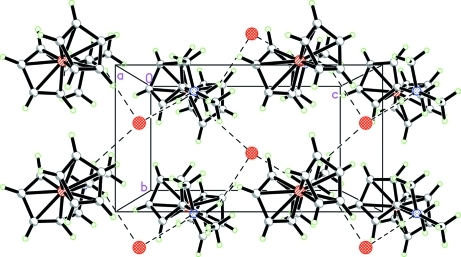
Crystal packing of the title compound viewed along the a axis showing the N+—H···Br- interactions (dotted line). Short C—H···Br- contacts are also indicated.

## supplementary crystallographic information

### Comment

A number of crystal structures containing the (*N*-ferrocenylmethyl)dimethylammonium cation, [Fe(C_5_H_5_)(C_8_H_13_N)]^+^, have been reported, including [Fe(C_5_H_5_)(C_8_H_13_N)]^+^Cl^-^.2H_2_O (Guo, Zhou *et al.*, 2006), 2[Fe(C_5_H_5_)(C_8_H_13_N)]^+^[ZnCl_4_]^2-^.H_2_O (Gibbons & Trotter, 1971), [Fe(C_5_H_5_)(C_8_H_13_N)]^+^NO_3_^-^ (Guo, Yang & Zhang, 2006) and 2[Fe(C_5_H_5_)(C_8_H_13_N)]^+^SO_4_^2-^.5H_2_O (Guo, 2006). The analogous chloride compound has also been reported (Winter & Wolmershauser, 1998) and the title compound is isomorphous with it.

The asymmetric unit consists of one (*N*-ferrocenylmethyl)dimethylammonium cation and one bromide anion (Fig. 1). The Fe atom is bonded to the two five-membered carbon rings with Fe—C bond lengths in the range 2.020 (6)-2.048 (7) Å, with mean values of 2.036 and 2.025Å for the unsubstituted and substituted Cp rings, respectively. The Fe···Cp plane distances are 1.638 and 1.651 Å for Cp1 and Cp2, respectively, and the Cp1—Fe—Cp2 angle is 178.58°. These suggest that an interaction may exist between the methyldimethylamine group and the Fe atom, drawing the less electron-rich substituted Cp ligand marginally closer to the metal centre. The two rings, Cp1(C1–C5) and Cp2 (C6–C10), are nearly parallel with a dihedral angle between their mean planes of 1.7°. They exhibit a nearly eclipsed conformation, as is usually found in other ferrocene derivatives (for example, the structures listed above).

In the crystal structure, the cations and bromide ions are connected by N^+^—H···Br^-^ hydrogen bonds (Fig. 2).

### Experimental

(Ferrocenylmethyl)dimethylamine (0.607 g, 2.5 mmol) was dissolved in ethanol (15 ml) and a yellow solid was obtained after adding HBr (0.5 g, 40%). The precipitate was dissolved by adding DMF and single crystals of the title compound suitable for X-ray analysis were obtained on slow evaporation of the solvents over a period of 7 days.

The dielectric constant of the compound as a function of temperature indicates that the permittivity is basically temperature-independent (ε = C/(T—T_0_)), suggesting that this compound is not ferroelectric or there may be no distinct phase transition occurring within the measured temperature range. Similarly, below the melting point of the compound (184°C), the dielectric constant as a function of temperature goes smoothly, and no dielectric anomaly is observed.

### Refinement

H atoms bound to C atoms were positioned geometrically, with C—H = 0.98, 0.97 and 0.96 Å for those on cyclopentadienyl, methylene and methyl C atoms, respectively, and refined as riding with *U*_iso_(H) = 1.2*U*_eq_(C) or 1.5*U*_eq _(methyl C). The methyl groups were allowed to rotate about their local threefold axes. Atom H1A was positioned geometrically and allowed to ride on N1, with N—H = 0.91 Å and *U*_iso_(H) = 1.2*U*_eq_(N).

### Crystal data

**Table:**

[Fe(C_5_H_5_)(C_8_H_13_N)]Br	*D*_x_ = 1.571 Mg m^−^^3^
*M**_r_* = 324.04	Melting point: 457 K
Orthorhombic, *P**n**a*2_1_	Mo *K*α radiation, λ = 0.71073 Å
Hall symbol: P 2c -2n	Cell parameters from 6130 reflections
*a* = 21.393 (4) Å	θ = 3.4–27.6°
*b* = 5.9296 (12) Å	µ = 3.99 mm^−^^1^
*c* = 10.798 (2) Å	*T* = 298 K
*V* = 1369.7 (5) Å^3^	Prism, yellow
*Z* = 4	0.20 × 0.20 × 0.20 mm
*F*(000) = 656	

### Data collection

**Table:**

Rigaku SCXmini diffractometer	3117 independent reflections
Radiation source: fine-focus sealed tube	2638 reflections with *I* > 2σ(*I*)
graphite	*R*_int_ = 0.056
ω scans	θ_max_ = 27.5°, θ_min_ = 3.6°
Absorption correction: multi-scan *CrystalClear* (Rigaku, 2005)	*h* = −27→27
*T*_min_ = 0.450, *T*_max_ = 0.468	*k* = −7→7
13217 measured reflections	*l* = −14→14

### Refinement

**Table:**

Refinement on *F*^2^	Secondary atom site location: difference Fourier map
Least-squares matrix: full	Hydrogen site location: inferred from neighbouring sites
*R*[*F*^2^ > 2σ(*F*^2^)] = 0.038	H-atom parameters constrained
*wR*(*F*^2^) = 0.078	*w* = 1/[σ^2^(*F*_o_^2^) + (0.0233*P*)^2^] where *P* = (*F*_o_^2^ + 2*F*_c_^2^)/3
*S* = 1.08	(Δ/σ)_max_ < 0.001
3117 reflections	Δρ_max_ = 0.28 e Å^−^^3^
147 parameters	Δρ_min_ = −0.51 e Å^−^^3^
1 restraint	Absolute structure: Flack (1983), 1474 Friedel pairs
Primary atom site location: structure-invariant direct methods	Flack parameter: 0.011 (12)

### Special details

**Table:**

Geometry. All esds (except the esd in the dihedral angle between two l.s. planes) are estimated using the full covariance matrix. The cell esds are taken into account individually in the estimation of esds in distances, angles and torsion angles; correlations between esds in cell parameters are only used when they are defined by crystal symmetry. An approximate (isotropic) treatment of cell esds is used for estimating esds involving l.s. planes.
Refinement. Refinement of F^2^ against ALL reflections. The weighted R-factor wR and goodness of fit S are based on F^2^, conventional R-factors R are based on F, with F set to zero for negative F^2^. The threshold expression of F^2^ > 2sigma(F^2^) is used only for calculating R-factors(gt) etc. and is not relevant to the choice of reflections for refinement. R-factors based on F^2^ are statistically about twice as large as those based on F, and R- factors based on ALL data will be even larger.

### Fractional atomic coordinates and isotropic or equivalent isotropic displacement parameters (Å^2^)

**Table:**

	*x*	*y*	*z*	*U*_iso_*/*U*_eq_	
Br1	0.56302 (2)	0.12331 (6)	0.47649 (4)	0.05861 (15)	
Fe1	0.31150 (2)	0.40436 (7)	0.28011 (5)	0.03551 (13)	
N1	0.51727 (12)	0.3869 (4)	0.2374 (3)	0.0333 (7)	
H1A	0.5230	0.3024	0.3069	0.040*	
C1	0.40534 (14)	0.4348 (5)	0.2980 (3)	0.0309 (7)	
C2	0.38089 (16)	0.2954 (6)	0.3942 (3)	0.0383 (8)	
H2A	0.3922	0.1379	0.4103	0.046*	
C3	0.33669 (18)	0.4253 (7)	0.4615 (4)	0.0472 (10)	
H3A	0.3119	0.3727	0.5321	0.057*	
C4	0.33448 (18)	0.6430 (6)	0.4089 (4)	0.0462 (10)	
H4A	0.3078	0.7677	0.4364	0.055*	
C5	0.37663 (15)	0.6481 (5)	0.3083 (3)	0.0351 (8)	
H5A	0.3843	0.7777	0.2541	0.042*	
C6	0.22327 (19)	0.2783 (10)	0.2675 (5)	0.0724 (14)	
H6A	0.1959	0.2412	0.3375	0.087*	
C7	0.22629 (19)	0.4831 (8)	0.2085 (4)	0.0591 (12)	
H7A	0.2016	0.6166	0.2302	0.071*	
C8	0.2691 (2)	0.4729 (9)	0.1167 (4)	0.0666 (13)	
H8A	0.2801	0.5962	0.0603	0.080*	
C9	0.2945 (2)	0.2549 (12)	0.1143 (6)	0.0845 (19)	
H9A	0.3258	0.1978	0.0560	0.101*	
C10	0.2658 (3)	0.1322 (7)	0.2113 (7)	0.090 (2)	
H10A	0.2732	−0.0262	0.2328	0.108*	
C11	0.44972 (15)	0.3637 (6)	0.2002 (4)	0.0357 (9)	
H11A	0.4424	0.4538	0.1266	0.043*	
H11B	0.4415	0.2074	0.1790	0.043*	
C12	0.55769 (16)	0.2923 (9)	0.1398 (4)	0.0611 (12)	
H12A	0.5456	0.1393	0.1235	0.092*	
H12B	0.6004	0.2960	0.1668	0.092*	
H12C	0.5533	0.3802	0.0657	0.092*	
C13	0.5351 (2)	0.6194 (5)	0.2675 (5)	0.0614 (12)	
H13A	0.5786	0.6242	0.2893	0.092*	
H13B	0.5104	0.6720	0.3360	0.092*	
H13C	0.5278	0.7143	0.1969	0.092*	

### Atomic displacement parameters (Å^2^)

**Table:**

	*U*^11^	*U*^22^	*U*^33^	*U*^12^	*U*^13^	*U*^23^
Br1	0.0834 (3)	0.0501 (2)	0.0424 (2)	0.0079 (2)	−0.0100 (2)	0.0080 (2)
Fe1	0.0313 (2)	0.0357 (3)	0.0395 (3)	−0.00206 (18)	−0.0070 (3)	−0.0036 (3)
N1	0.0348 (16)	0.0343 (16)	0.0308 (16)	0.0011 (11)	−0.0007 (12)	0.0048 (12)
C1	0.0279 (15)	0.0322 (16)	0.032 (2)	−0.0026 (13)	−0.0029 (15)	−0.0051 (15)
C2	0.036 (2)	0.039 (2)	0.040 (2)	−0.0068 (16)	−0.0095 (17)	0.0043 (17)
C3	0.037 (2)	0.072 (3)	0.033 (2)	−0.0095 (18)	0.0045 (18)	0.000 (2)
C4	0.037 (2)	0.051 (3)	0.050 (2)	0.0009 (16)	−0.0006 (19)	−0.0205 (19)
C5	0.0353 (18)	0.0278 (18)	0.042 (2)	−0.0027 (12)	−0.0022 (16)	−0.0036 (15)
C6	0.040 (2)	0.103 (4)	0.074 (4)	−0.030 (3)	−0.024 (3)	0.007 (3)
C7	0.046 (2)	0.063 (3)	0.068 (3)	0.013 (2)	−0.023 (2)	−0.007 (3)
C8	0.068 (3)	0.080 (4)	0.051 (3)	−0.008 (3)	−0.024 (2)	0.008 (3)
C9	0.049 (3)	0.128 (5)	0.076 (4)	0.016 (3)	−0.031 (3)	−0.064 (4)
C10	0.091 (4)	0.034 (3)	0.145 (6)	−0.013 (3)	−0.078 (4)	−0.002 (3)
C11	0.0324 (19)	0.040 (2)	0.035 (2)	0.0025 (14)	−0.0033 (15)	−0.0050 (15)
C12	0.042 (2)	0.089 (3)	0.053 (3)	0.000 (2)	0.016 (2)	−0.001 (3)
C13	0.054 (2)	0.054 (3)	0.077 (4)	−0.0162 (17)	−0.010 (3)	0.003 (3)

### Geometric parameters (Å, °)

**Table:**

Fe1—C1	2.025 (3)	C4—C5	1.412 (5)
Fe1—C8	2.025 (4)	C4—H4A	0.980
Fe1—C10	2.029 (4)	C5—H5A	0.980
Fe1—C5	2.031 (3)	C6—C7	1.373 (7)
Fe1—C9	2.031 (5)	C6—C10	1.395 (8)
Fe1—C7	2.034 (4)	C6—H6A	0.980
Fe1—C2	2.034 (3)	C7—C8	1.351 (6)
Fe1—C6	2.035 (4)	C7—H7A	0.980
Fe1—C3	2.035 (4)	C8—C9	1.402 (7)
Fe1—C4	2.044 (4)	C8—H8A	0.980
N1—C13	1.467 (4)	C9—C10	1.416 (8)
N1—C12	1.474 (5)	C9—H9A	0.980
N1—C11	1.506 (4)	C10—H10A	0.980
N1—H1A	0.910	C11—H11A	0.970
C1—C5	1.410 (4)	C11—H11B	0.970
C1—C2	1.427 (5)	C12—H12A	0.960
C1—C11	1.482 (5)	C12—H12B	0.960
C2—C3	1.420 (5)	C12—H12C	0.960
C2—H2A	0.980	C13—H13A	0.960
C3—C4	1.411 (5)	C13—H13B	0.960
C3—H3A	0.980	C13—H13C	0.960
			
C1—Fe1—C8	120.62 (19)	C2—C3—Fe1	69.5 (2)
C1—Fe1—C10	125.8 (2)	C4—C3—H3A	125.9
C8—Fe1—C10	67.9 (2)	C2—C3—H3A	125.9
C1—Fe1—C5	40.70 (12)	Fe1—C3—H3A	125.9
C8—Fe1—C5	107.17 (17)	C3—C4—C5	107.9 (3)
C10—Fe1—C5	162.2 (3)	C3—C4—Fe1	69.4 (2)
C1—Fe1—C9	107.51 (17)	C5—C4—Fe1	69.2 (2)
C8—Fe1—C9	40.4 (2)	C3—C4—H4A	126.0
C10—Fe1—C9	40.8 (2)	C5—C4—H4A	126.0
C5—Fe1—C9	124.5 (2)	Fe1—C4—H4A	126.0
C1—Fe1—C7	154.72 (18)	C1—C5—C4	108.6 (3)
C8—Fe1—C7	38.89 (18)	C1—C5—Fe1	69.44 (16)
C10—Fe1—C7	67.12 (19)	C4—C5—Fe1	70.24 (19)
C5—Fe1—C7	120.56 (16)	C1—C5—H5A	125.7
C9—Fe1—C7	66.71 (19)	C4—C5—H5A	125.7
C1—Fe1—C2	41.15 (14)	Fe1—C5—H5A	125.7
C8—Fe1—C2	156.64 (19)	C7—C6—C10	108.5 (5)
C10—Fe1—C2	108.73 (17)	C7—C6—Fe1	70.3 (2)
C5—Fe1—C2	68.57 (13)	C10—C6—Fe1	69.7 (2)
C9—Fe1—C2	121.73 (19)	C7—C6—H6A	125.8
C7—Fe1—C2	163.11 (19)	C10—C6—H6A	125.8
C1—Fe1—C6	163.51 (19)	Fe1—C6—H6A	125.8
C8—Fe1—C6	66.4 (2)	C8—C7—C6	109.4 (5)
C10—Fe1—C6	40.2 (2)	C8—C7—Fe1	70.2 (2)
C5—Fe1—C6	155.2 (2)	C6—C7—Fe1	70.3 (2)
C9—Fe1—C6	67.3 (2)	C8—C7—H7A	125.3
C7—Fe1—C6	39.44 (19)	C6—C7—H7A	125.3
C2—Fe1—C6	126.91 (19)	Fe1—C7—H7A	125.3
C1—Fe1—C3	68.90 (15)	C7—C8—C9	108.5 (5)
C8—Fe1—C3	160.9 (2)	C7—C8—Fe1	70.9 (3)
C10—Fe1—C3	121.9 (2)	C9—C8—Fe1	70.0 (3)
C5—Fe1—C3	68.31 (15)	C7—C8—H8A	125.7
C9—Fe1—C3	157.3 (2)	C9—C8—H8A	125.7
C7—Fe1—C3	126.08 (18)	Fe1—C8—H8A	125.7
C2—Fe1—C3	40.84 (15)	C8—C9—C10	107.0 (5)
C6—Fe1—C3	109.4 (2)	C8—C9—Fe1	69.6 (3)
C1—Fe1—C4	68.57 (14)	C10—C9—Fe1	69.5 (3)
C8—Fe1—C4	124.18 (19)	C8—C9—H9A	126.5
C10—Fe1—C4	156.4 (3)	C10—C9—H9A	126.5
C5—Fe1—C4	40.55 (15)	Fe1—C9—H9A	126.5
C9—Fe1—C4	160.9 (3)	C6—C10—C9	106.6 (4)
C7—Fe1—C4	108.37 (17)	C6—C10—Fe1	70.1 (3)
C2—Fe1—C4	68.43 (15)	C9—C10—Fe1	69.7 (2)
C6—Fe1—C4	121.5 (2)	C6—C10—H10A	126.7
C3—Fe1—C4	40.46 (15)	C9—C10—H10A	126.7
C13—N1—C12	111.3 (3)	Fe1—C10—H10A	126.7
C13—N1—C11	113.2 (3)	C1—C11—N1	113.4 (3)
C12—N1—C11	109.7 (3)	C1—C11—H11A	108.9
C13—N1—H1A	107.5	N1—C11—H11A	108.9
C12—N1—H1A	107.5	C1—C11—H11B	108.9
C11—N1—H1A	107.5	N1—C11—H11B	108.9
C5—C1—C2	107.6 (3)	H11A—C11—H11B	107.7
C5—C1—C11	126.2 (3)	N1—C12—H12A	109.5
C2—C1—C11	126.1 (3)	N1—C12—H12B	109.5
C5—C1—Fe1	69.86 (18)	H12A—C12—H12B	109.5
C2—C1—Fe1	69.78 (18)	N1—C12—H12C	109.5
C11—C1—Fe1	122.8 (2)	H12A—C12—H12C	109.5
C3—C2—C1	107.6 (3)	H12B—C12—H12C	109.5
C3—C2—Fe1	69.6 (2)	N1—C13—H13A	109.5
C1—C2—Fe1	69.07 (19)	N1—C13—H13B	109.5
C3—C2—H2A	126.2	H13A—C13—H13B	109.5
C1—C2—H2A	126.2	N1—C13—H13C	109.5
Fe1—C2—H2A	126.2	H13A—C13—H13C	109.5
C4—C3—C2	108.2 (3)	H13B—C13—H13C	109.5
C4—C3—Fe1	70.1 (2)		

### Hydrogen-bond geometry (Å, °)

**Table:**

*D*—H···*A*	*D*—H	H···*A*	*D*···*A*	*D*—H···*A*
N1—H1A···Br1	0.91	2.28	3.172 (3)	165
